# Porcine Epidemic Diarrhea Virus Antagonizes Host IFN-λ-Mediated Responses by Tilting Transcription Factor STAT1 toward Acetylation over Phosphorylation To Block Its Activation

**DOI:** 10.1128/mbio.03408-22

**Published:** 2023-04-13

**Authors:** Jidong Xu, Qin Gao, Weiwu Zhang, Jingyou Zheng, Rong Chen, Xiao Han, Junyong Mao, Ying Shan, Fushan Shi, Fang He, Weihuan Fang, Xiaoliang Li

**Affiliations:** a Department of Veterinary Medicine, College of Animal Sciences, Institute of Preventive Veterinary Medicine, Zhejiang University, Hangzhou, Zhejiang, China; b Zhejiang Province Key Laboratory of Veterinary Medicine, MOA Key Laboratory of Animal Virology, Zhejiang University, Hangzhou, Zhejiang, China; c Hangzhou Academy of Agricultural Sciences, Hangzhou, Zhejiang, China; d Yongyou Industry Park, Yazhou Bay Sci-Tech City, Sanya, China; Griffith University-Gold Coast Campus

**Keywords:** porcine epidemic diarrhea virus, signal transducer and activator of transcription 1, histone deacetylase 1, acetylation, interferon-stimulated genes

## Abstract

Porcine epidemic diarrhea virus (PEDV) is the main etiologic agent causing acute swine epidemic diarrhea, leading to severe economic losses to the pig industry. PEDV has evolved to deploy complicated antagonistic strategies to escape from host antiviral innate immunity. Our previous study demonstrated that PEDV downregulates histone deacetylase 1 (HDAC1) expression by binding viral nucleocapsid (N) protein to the transcription factor Sp1, inducing enhanced protein acetylation. We hypothesized that PEDV inhibition of HDAC1 expression would enhance acetylation of the molecules critical in innate immune signaling. Signal transducer and activator of transcription 1 (STAT1) is a crucial transcription factor regulating expression of interferon (IFN)-stimulated genes (ISGs) and anti-PEDV immune responses, as shown by overexpression, chemical inhibition, and gene knockdown in IPEC-J2 cells. We further show that PEDV infection and its N protein overexpression, although they upregulated STAT1 transcription level, could significantly block poly(I·C) and IFN-λ3-induced STAT1 phosphorylation and nuclear localization. Western blotting revealed that PEDV and its N protein promote STAT1 acetylation via downregulation of HDAC1. Enhanced STAT1 acetylation due to HDAC1 inhibition by PEDV or MS-275 (an HDAC1 inhibitor) impaired STAT1 phosphorylation, indicating that STAT1 acetylation negatively regulated its activation. These results, together with our recent report on PEDV N-mediated inhibition of Sp1, clearly indicate that PEDV manipulates the Sp1-HDAC1-STAT1 signaling axis to inhibit transcription of *OAS1* and *ISG15* in favor of its replication. This novel immune evasion mechanism is realized by suppression of STAT1 activation through preferential modulation of STAT1 acetylation over phosphorylation as a result of HDAC1 expression inhibition.

## INTRODUCTION

Porcine epidemic diarrhea virus (PEDV) is a positive-stranded RNA virus belonging to the genus *Alphacoronavirus* of the family *Coronaviridae* (1). It is the causative agent of porcine epidemic diarrhea (PED), an acute intestinal disease in suckling piglets shown as severe diarrhea and dehydration (2). Since its initial outbreaks in European countries in the 1970s, PED has spread worldwide and brought huge damage to the swine industry in many countries (3, 4).

The PEDV genome is approximately 28,000 nucleotides (nt) in length, encoding 5 structural proteins, spike protein (S protein), open reading frame 3 (ORF3), envelope protein (E protein), membrane protein (M protein), and nucleocapsid protein (N protein) from the 5′-to-3′ order and 16 nonstructural proteins (5). Similar to other RNA viruses, PEDV exhibits a high mutation rate (6) and has developed sophisticated immune evasion mechanisms by means of its structural and nonstructural proteins (7). The PEDV N protein is not only involved in viral RNA replication and transcription (8) but also acts as an important viral component to antagonize host interferon (IFN) signaling by sequestering the interaction of interferon regulatory factor 3 (IRF3) with TBK1 (9). The PEDV S protein supports viral adsorption to permissive cells and subsequent invasion (10), and its S1 domain induces apoptosis (11). The nonstructural proteins have also been found to evade host innate immune responses. Zhang et al. reported that nsp1 blocks IκBα phosphorylation, resulting in inhibition of p65 activation and tumor necrosis factor alpha (TNF-α) production, and a number of other PEDV proteins, such as E protein, ORF3, as well as nsp3, -5, -7, -14, -15, and -16, have also been shown to suppress NF-κB activation during PEDV infection (12).

As one of the most important cytokines secreted by the immune cells, IFNs display intense antiviral activity (13, 14). Specific receptors, such as IFNAR1-IFNAR2 and IL-28R1-IL-28R2 complexes, recognize the extracellular IFNs and activate the downstream signal transducer and activator of transcription (STAT) signaling pathway (15). Type III IFNs (IFN-λs) are mainly expressed in epithelial cells (16, 17) and activate the downstream antiviral immune responses through JAK-STAT signaling (18). Classical positive feedback regulation of IFN signaling describes that the secretory IFN-λ induced by virus infection or poly(I·C) stimulation binds to specific membrane receptors, IL-28R1 and IL-28R2, followed by activating JAK1 and Tyk2 proteins, which, in turn, induces STAT1 and STAT2 phosphorylation (17, 19). The activated STAT proteins then form a heterodimer and interact with another integral transcription factor, IRF9 (20). The STATs-IRF9 transcription factor complex, also known as interferon-stimulated gene factor 3 (ISGF3), finally translocates into the nucleus and activates transcription of ISGs (21, 22). Because the ISGs, such as ISG15, OAS, IFITM3, and MX1, display strong antiviral activity, STAT proteins are indicated as crucial antiviral regulators (23, 24).

Our initial experiments revealed that STAT1 overexpression strongly inhibits PEDV infection. Interestingly, we found that STAT1 activation is diminished, while its transcription level is highly induced in the PEDV-infected IPEC-J2 cells. The contradictory phenomenon drove us to study the interaction of PEDV and STAT1. Our previous study found that PEDV utilizes its N protein to bind transcription factor Sp1, inhibiting transcription of HDAC1 and expression of some ISGs, and downregulation of HDAC1 by PEDV enhances histone acetylation (25). We, therefore, asked whether PEDV N protein-mediated inhibition of HDAC1 affects acetylation and activation of STAT1, which further influences expression of ISGs. We discovered that PEDV induced STAT1 acetylation in IPEC-J2 cells at a level similar to HDAC1 knockdown or treatment with MS-275 (an HDAC1- and HDAC3-specific inhibitor) and that PEDV infection prevented poly(I·C)-induced phosphorylation of STAT1 and its nuclear translocation. Further experiments revealed that the N protein prevented STAT1 phosphorylation induced by IFN-λ3, but with concurrent increase of STAT1 acetylation. Subsequently, the impaired STAT1 activation leads to downregulation of antiviral ISGs (*ISG15* and *OAS1*). Our study revealed a novel immune evasion mechanism that PEDV uses its N protein to block STAT1 activation and its downstream initiation of ISG expression by induction of STAT1 acetylation over phosphorylation. Increased STAT1 acetylation results from HDAC1 downregulation as a result of N protein-mediated sequestration of Sp1 from its transcriptional activity, as we have recently reported (25).

## RESULTS

### PEDV inhibits IFN agonist-induced upregulation of ISGs in IPEC-J2 cells.

**FIG 1:**
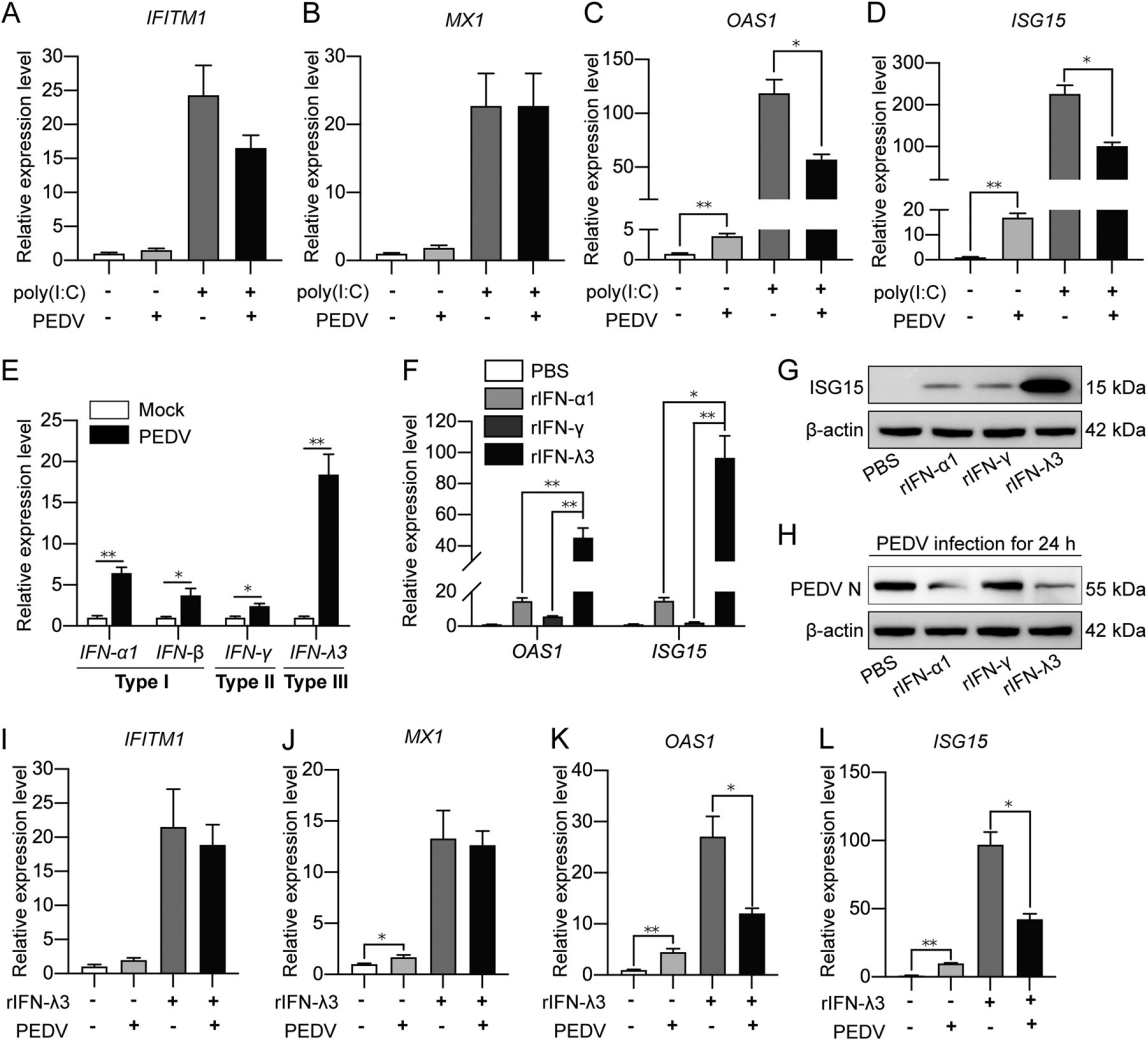
PEDV impairs poly(I·C)- and rIFN-λ3-induced transcription of *OAS1* and *ISG15* in IPEC-J2 cells. The IPEC-J2 cells were transfected with 0.1 μg/mL of poly(I·C) for 12 h and then infected with PEDV (MOI = 1) for 24 h. The cells were collected for extraction of total mRNA and were used to detect transcription of the ISGs *IFITM1* (A), *MX1* (B), *OAS1* (C), and *ISG15* (D). (E) Transcriptional changes of type I to III IFNs after PEDV infection for 24 h detected by qRT-PCR. (F and G) The IPEC-J2 cells were treated with recombinant IFN-α1, IFN-γ, and IFN-λ3 at the concentration of 50 ng/mL for 24 h, after which total mRNA was extracted for detecting *OAS1* and *ISG15* transcription level by qRT-PCR (F), and the whole-cell lysate was used for detecting ISG15 expression by Western blotting (G). (H) The cells were treated with recombinant IFN-α1, IFN-γ, and IFN-λ3 for 30 min as mentioned above, followed by PEDV infection; PEDV N expression level was detected by Western blotting after PEDV infection for 24 h. (I to L) IPEC-J2 cells were pretreated with rIFN-λ3 at the concentration of 50 ng/mL, followed by PEDV infection at the MOI of 1. Total RNA was extracted at 24 h post-PEDV infection. The transcription levels of *IFITM1*, *MX1*, *OAS1*, and *ISG15* were detected by qRT-PCR. Transcription in the control cells without poly(I·C) or rIFN-λ3 treatment and PEDV infection was normalized to the housekeeping gene, *GAPDH*. The results were shown as means ± SD from three independent experiments. *, *P < *0.05; **, *P < *0.01.

To examine the relationship between PEDV and host IFN signaling, we transfected the IPEC-J2 cells with poly(I·C) followed by PEDV infection to analyze the transcriptional levels of different ISGs, *IFITM1*, *MX1*, *OAS1*, and *ISG15*. As shown in Fig. 1A to D, all ISG genes were upregulated by poly(I·C) in IPEC-J2 cells, indicating that poly(I·C) induces strong activation of the IFN signaling. Intriguingly, PEDV infection, though leading to mild upregulation of the transcription of ISG genes, significantly impaired poly(I·C)-induced expression of *OAS1* and *ISG15* (Fig. 1C and D). It is known that the secreted type III interferon (IFN-λ) strongly stimulates JAK-STAT1 signaling in mucosal defense (26). Figure 1E clearly shows that infection of the IPEC-J2 cells with the PEDV induced a much higher level of IFN-λ3 transcription than the type I IFNs (IFN-α and -β) and type II IFN (IFN-γ). This is consistent with the recent studies showing predominant IFN-λ responses in IPEC-J2 cells over type I IFNs (16, 27). Therefore, it is evident that type III IFN signaling plays the main role in IPEC-J2 cells and anti-PEDV innate immune responses. To avoid sequential induction of IFNs and ISGs by poly(I·C) as a pan-activator, we treated the IPEC-J2 cells with different recombinant IFNs (rIFNs; rIFN-α1, rIFN-γ, and rIFN-λ3) to examine their effects on expression of *OAS1* and ISG15 as well as PEDV replication. Figure 1F to H reveals that rIFN-λ3 induced much higher expression of *OAS1* and *ISG15* than rIFN-α1 and rIFN-γ, while both rIFN-λ3 and rIFN-α1 inhibited PEDV replication (Fig. 1F to H). Consequently, we focused on PEDV infection in affecting IFN-λ signaling in IPEC-J2 cells. rIFN-λ3 induced high expression of different antiviral ISGs (*IFITM1*, *MX1*, *OAS1*, and *ISG15*), while PEDV significantly inhibited rIFN-λ3-induced expression of *OAS1* and *ISG15* (Fig. 1I to L). The above-described results indicate that PEDV suppresses both poly(I·C)- and IFN-λ-mediated signaling, suggesting that the suppressive function might lie downstream of the IFN receptors, such as JAK-STAT signaling.

### PEDV upregulates STAT1 transcription in IPEC-J2 cells.

**FIG 2:**
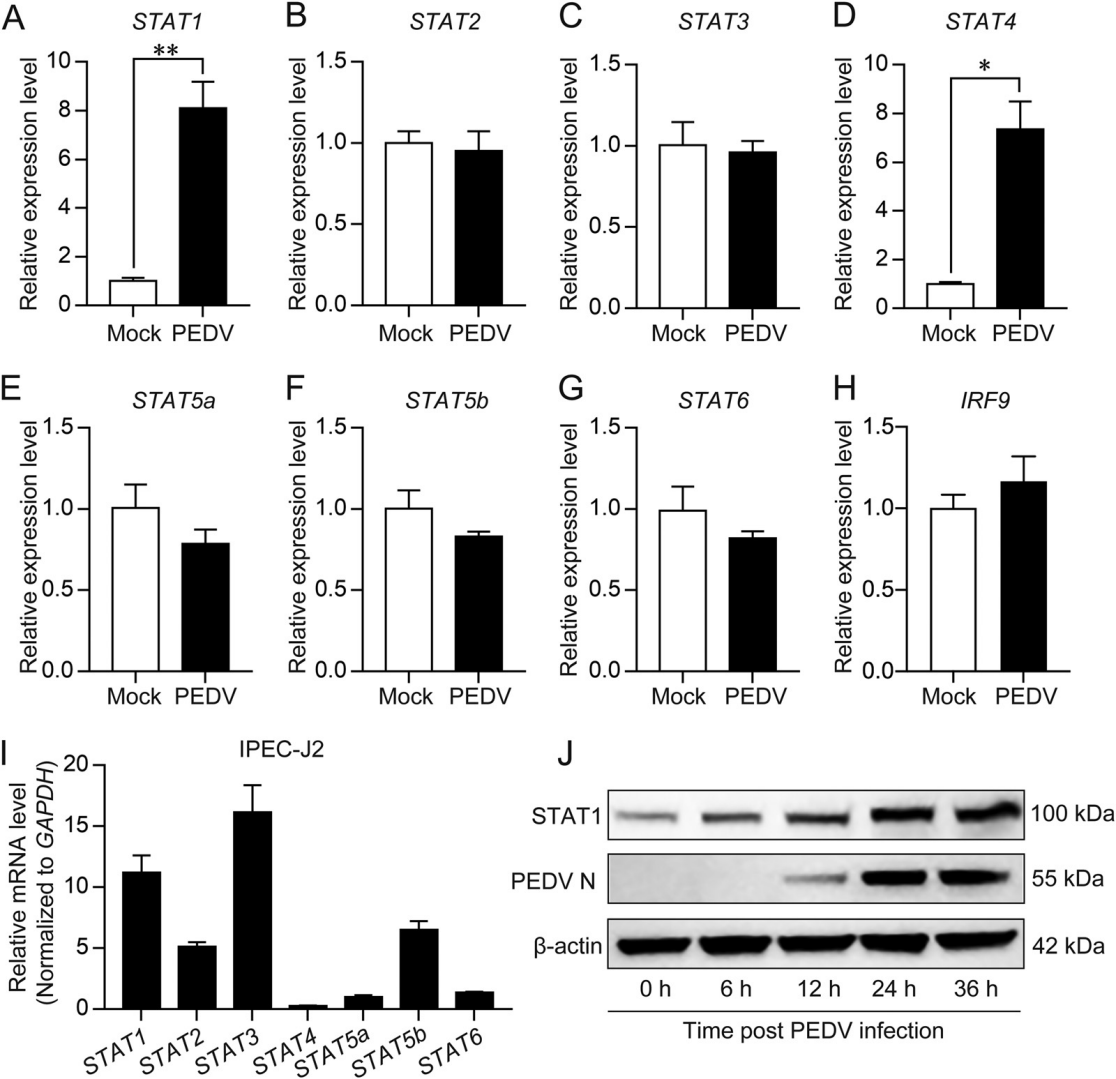
Expression analysis of different STATs in PEDV-infected IPEC-J2 cells. (A to H) Transcription of different STAT genes (*STAT1*, *STAT2*, *STAT3*, *STAT4*, *STAT5a*, *STAT5b*, and *STAT6*) and *IRF9* in IPEC-J2 cells infected with PEDV for 24 h detected by qRT-PCR. The uninfected cells with the same amount of trypsin treatment only were used as the mock control. The data were shown as means ± SD of three independent experiments. *, *P < *0.05; **, *P < *0.01. (I) Expression profiles of different *STAT* genes in uninfected IPEC-J2 cells by qRT-PCR, the *STAT* genes were normalized to *GAPDH*, and the data are shown as means ± SD of three independent experiments. (J) Expression of STAT1 at different time points after PEDV infection detected by Western blotting. PEDV replication was represented by its N protein expression, and β-actin was used as a loading control.

Our recent study revealed that HDAC1 functions as an antiviral regulator against PEDV infection by activating IFN signaling (25), probably through the JAK-STAT pathway (28). To investigate whether PEDV infection would affect expression of STAT genes, the IPEC-J2 cells infected with PEDV were used to examine their expression by reverse transcription-quantitative PCR (qRT-PCR). Figure 2 shows that PEDV infection upregulated transcription of both *STAT1* and *STAT4* but did not have an apparent effect on other STAT genes (Fig. 2A to G), nor on *IRF9* (Fig. 2H), whose product (IRF9) forms the ISGF3 with STAT1 and STAT2 for nuclear transcription regulation (29). Since *STAT4* abundance is relatively low in unstimulated IPEC-J2 cells (Fig. 2I), we targeted STAT1 to study its function in PEDV infection. As shown in Fig. 2J, STAT1 was upregulated with the progression of PEDV infection.

### Porcine STAT1 functions as an antiviral regulator against PEDV infection.

**FIG 3:**
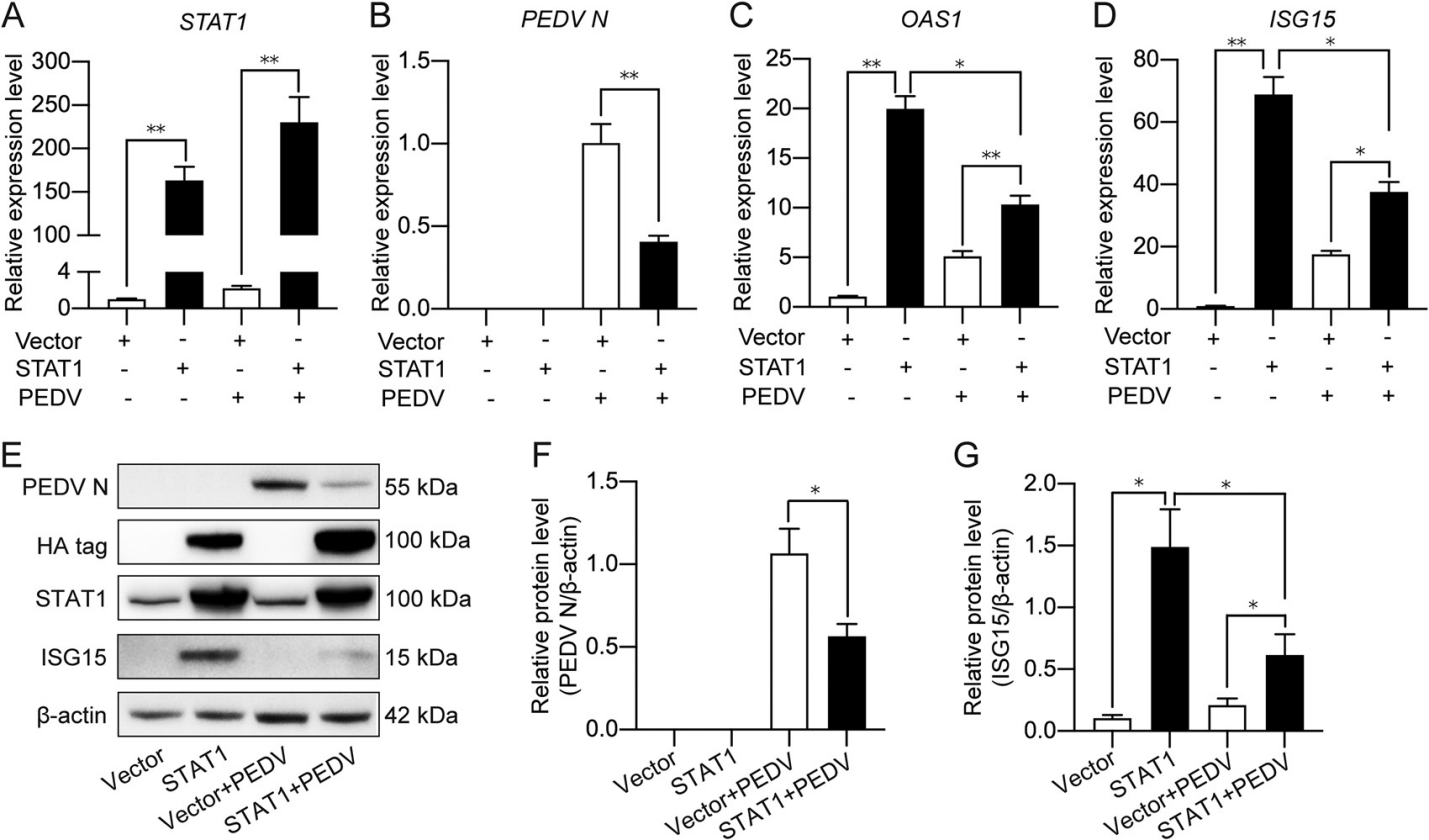
STAT1 overexpression inhibits PEDV replication and promotes ISG expression. The IPEC-J2 cells were transfected with pCMV-HA-STAT1 followed by PEDV infection for 24 h. Control vector-transfected cells or PEDV-infected cells were used as controls. Total RNA was extracted for detecting *STAT1* (A), PEDV *N* (B), *OAS1* (C), and *ISG15* (D) transcription by qRT-PCR. (E) PEDV N, STAT1, and ISG15 expression detected by Western blotting. Density analysis shown as relative PEDV N (F) and ISG15 (G) expression to β-actin were obtained from 3 independent experiments of (E). Results of panels A, B, C, D, F, and G are shown as means ± SD. *, *P < *0.05; **, *P < *0.01.

To examine whether STAT1 is involved in PEDV replication, STAT1 with a hemagglutinin (HA) tag was ectopically expressed in IPEC-J2 cells (Fig. 3A and E). As is indicated in Fig. 3B and F, STAT1 overexpression significantly repressed PEDV replication, shown as reduced N protein expression, possibly by increased transcription of *OAS1* and *ISG15* (Fig. 3C, D, and G). However, PEDV infection could effectively antagonize STAT1-induced expression of *OAS1* and *ISG15*. The above-described results indicate that PEDV, though negatively regulated by STAT1, could suppress STAT1-mediated ISG expression.

**FIG 4:**
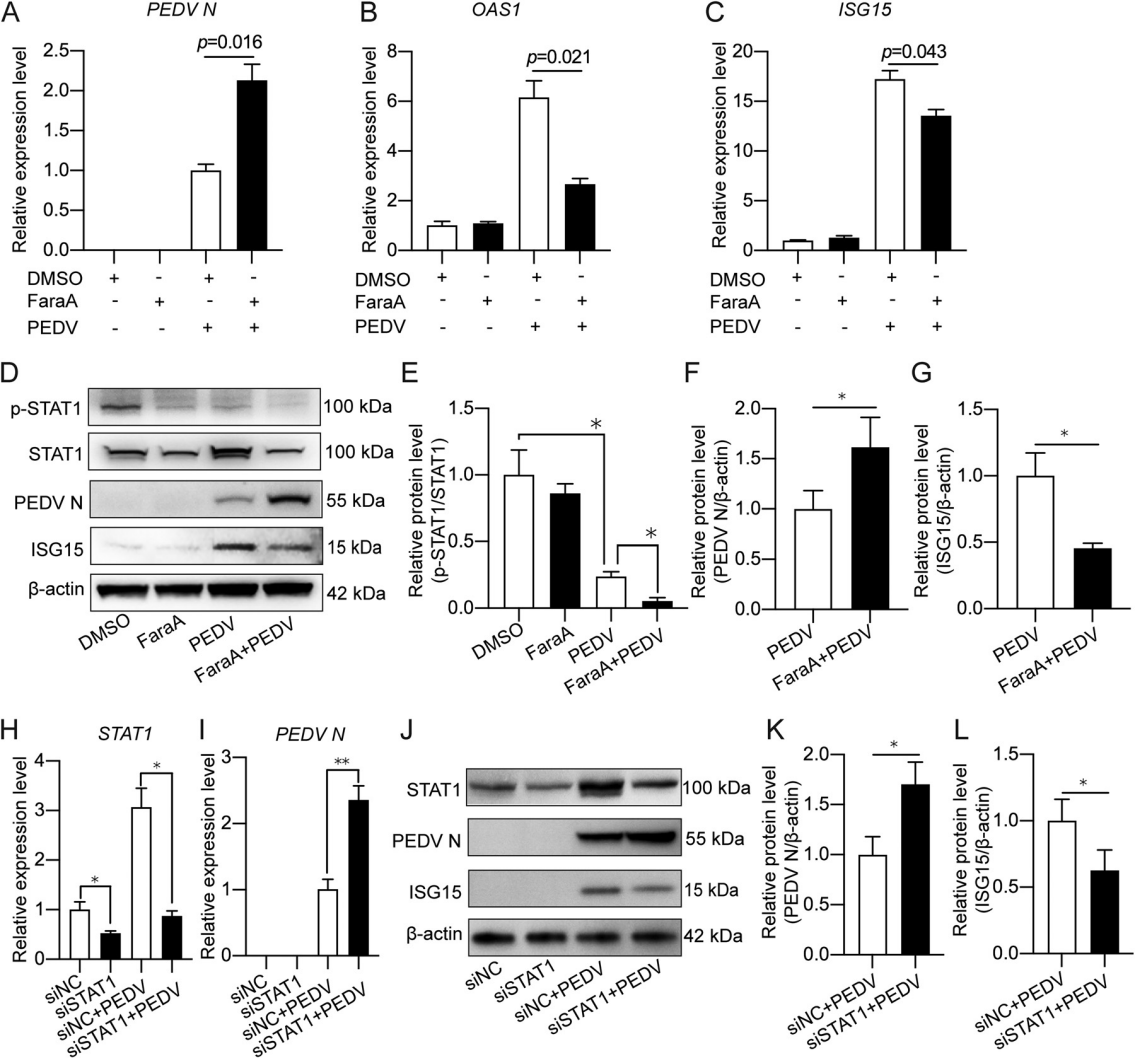
STAT1 inhibition promotes PEDV replication via downregulating ISG expression. (A to C) The IPEC-J2 cells were pretreated with 1 μM FaraA followed by PEDV infection (MOI = 1) for 24 h. Total RNA was extracted for transcriptional analysis of PEDV N, *OAS1*, and *ISG15* by qRT-PCR. Dimethyl sulfoxide (DMSO) treatment was used as a solvent control. (D) The same samples were also used for Western blotting to detect ISG15, PEDV N, total STAT1, and phosphorylated STAT1. (E to G) Densitometric analysis of p-STAT1/total STAT1, PEDV N/β-actin, and ISG15/β-actin of panel D. (H to L) The IPEC-J2 cells were transfected with STAT1 siRNA for 24 h followed by PEDV infection for qRT-PCR and Western blotting. (H) STAT1 knockdown efficiency detected by qPCR. (I) PEDV N transcription in STAT1 knockdown IPEC-J2 cells. (J) PEDV N, ISG15, and STAT1 expression in STAT1 knockdown and PEDV-infected cells detected by Western blotting. Density analysis shown as relative PEDV N (K) and ISG15 (L) expression to β-actin were obtained from 3 independent experiments of panel J. The results are shown as means ± SD from three independent experiments for panels A to C, E to G, H, and I. *, *P < *0.05; **, *P < *0.01.

Furthermore, chemical inhibition and RNA interference (RNAi) of STAT1 were performed to confirm its antiviral function. FaraA is reported as a selective STAT1 inhibitor by specifically depleting STAT1 mRNA and protein (30). The qPCR results showed that STAT1 inhibition by FaraA treatment in IPEC-J2 cells led to increased transcription of the PEDV N gene (Fig. 4A) and reduced expression of *OAS1* and *ISG15* (Fig. 4B, C, and G). Immunoblotting also revealed that FaraA treatment was effective in suppressing expression and phosphorylation of STAT1 and promoting PEDV replication (Fig. 4D to F). Similar findings were seen by STAT1 knockdown (Fig. 4H to L). Thus, STAT1 plays an anti-PEDV role by activating ISGs.

### PEDV infection suppresses STAT1 activation.

**FIG 5:**
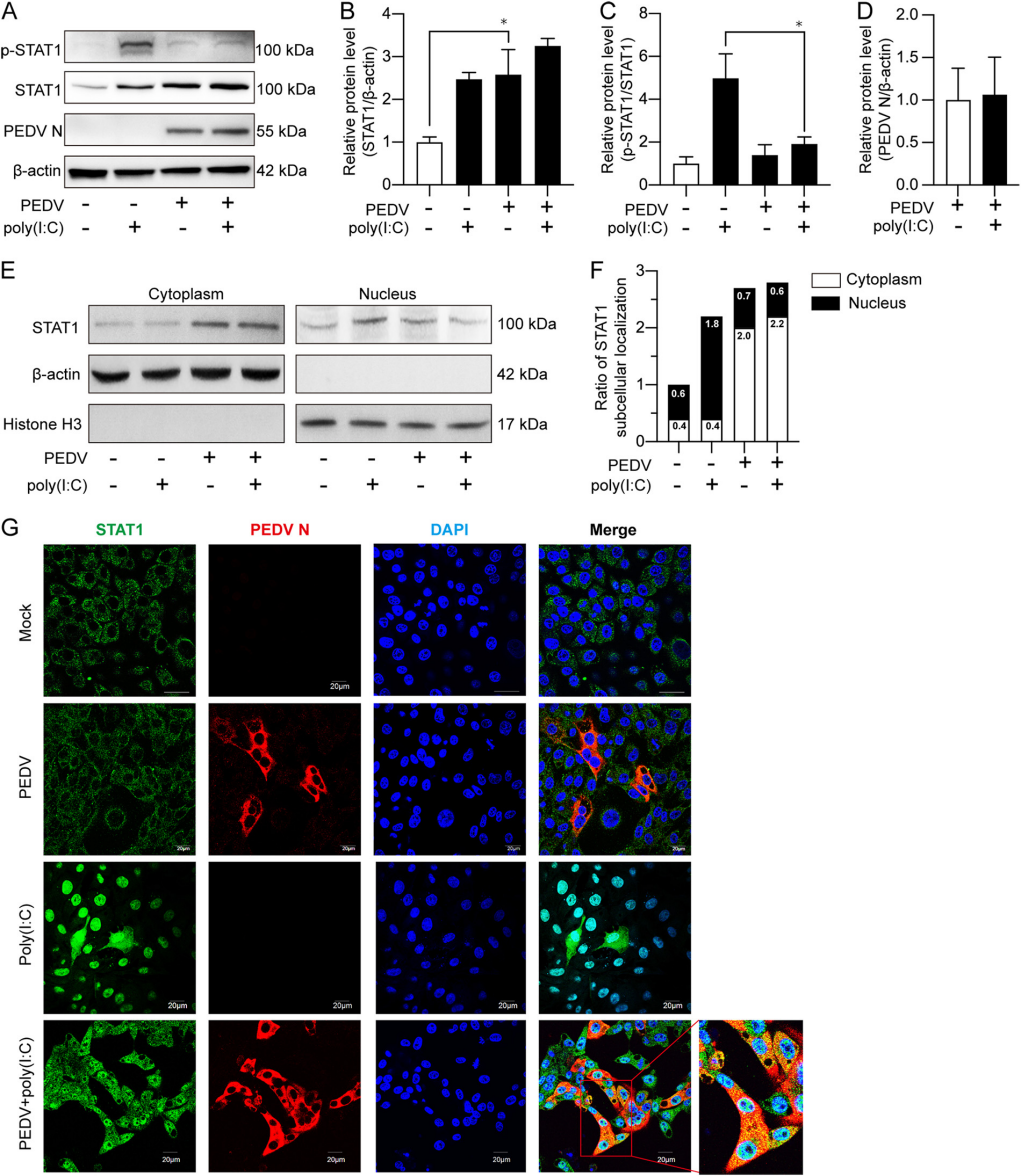
PEDV blocks STAT1 phosphorylation and translocation into the nuclei. (A) Changes of STAT1 phosphorylation in poly(I·C)-transfected and PEDV-infected IPEC-J2 cells. IPEC-J2 cells were inoculated with PEDV at the MOI of 1 for 4 h adsorption, after which poly(I·C) was transfected to the final concentration of 0.1 μg/mL for the next 20 h; poly(I·C) transfection or PEDV infection alone were used as controls, respectively. Total cell lysates were used to detect the expression of target proteins with β-actin as the loading control. (B to D) STAT1 expression, phosphorylation, and PEDV N expression as shown in panel A were analyzed and represented by STAT1/β-actin ratio (B), pSTAT1/STAT1 ratio (C), and PEDV N/β-actin ratio (D) based on 3 independent experiments, respectively. (E) STAT1 levels in the cytoplasmic and nuclear compartments as determined by Western blotting. (F) Ratios of STAT1 in the two compartments to total STAT1 in the whole-cell lysates based on panel E. Percentage of nuclear STAT1 is shown on top of the bars. The IPEC-J2 cells were treated as in panel A; β-actin and histone H3 were used as cytoplasmic and nuclear markers separately. (G) STAT1 subcellular localization in the cells treated as described above was also detected by immunocytochemistry with a confocal microscope; endogenous STAT1 and PEDV N were severally probed by rabbit anti-STAT1 and mouse anti-PEDV N antibodies. The nuclei were stained with DAPI.

We found that PEDV infection upregulated STAT1 expression (Fig. 2A and J) but seemed to suppress its phosphorylation (Fig. 4D), suggesting the possibility of viral suppression of STAT1 activation, an event more important than a mere increase of the total STAT1 in signaling. To establish if PEDV restrains STAT1 activation, poly(I·C) transfection was carried out along with PEDV infection in IPEC-J2 cells. As shown in Fig. 5A to D, poly(I·C) induced a high STAT1 phosphorylation level, whereas PEDV led to significant downregulation of poly(I·C)-induced STAT1 phosphorylation. Since phosphorylation is critical for nuclear importation of transcription factors (31), we analyzed the subcellular localization of STAT1 by nuclear and cytoplasmic fractionation. We found that the STAT1 fraction in the nuclei was significantly lower in PEDV-infected cells than in poly(I·C)-treated control cells (Fig. 5E and F), which is accordant with a decreased level of STAT1 phosphorylation (Fig. 4D and E). Immunofluorescence showed that STAT1 was localized in the cytoplasm of mock-infected or PEDV-infected cells and that PEDV infection effectively prevented poly(I·C)-induced nuclear accumulation of STAT1 (3rd-row panels versus 4th-row panels of Fig. 5G). These results clearly indicate that PEDV blocks poly(I·C)-induced phosphorylation and nuclear localization of STAT1, thus inhibiting its transcriptional activity of ISG genes.

### PEDV enhances STAT1 acetylation through inhibition of HDAC1 activity.

**FIG 6:**
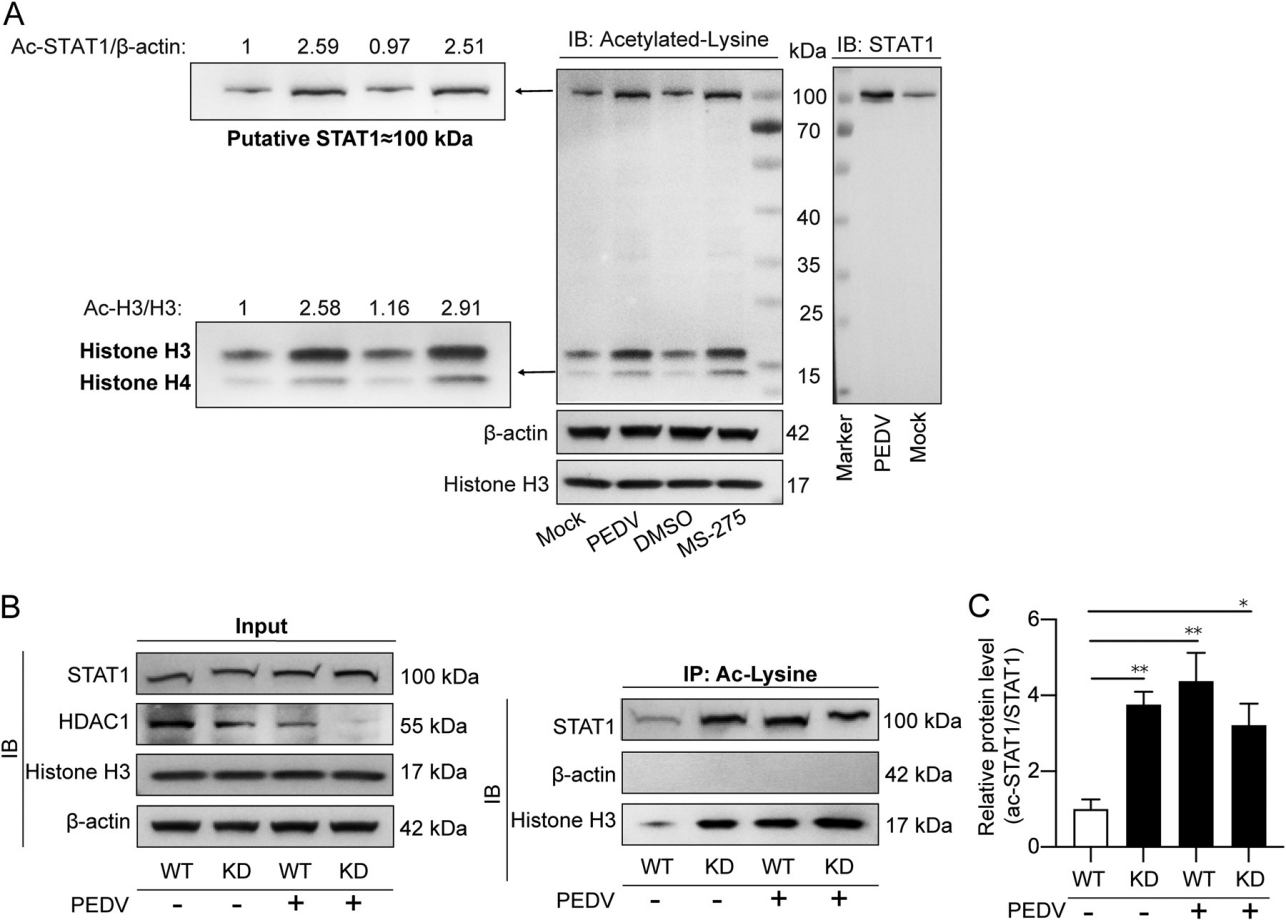
PEDV infection enhances significant STAT1 acetylation via inhibition of HDAC1 activity. (A) Western blotting detecting acetylated proteins in IPEC-J2 cells infected with PEDV or treated with MS-275. The IPEC-J2 cells were infected with PEDV at an MOI of 1 or treated with 1 μM MS-275 for 24 h. Total cell lysates were extracted for Western blotting using the acetyl-lysine antibody. Histone H3 and β-actin were used as loading controls. (B) STAT1 acetylation analyzed by co-IP using an acetyl-lysine antibody. Wild-type (WT) and HDAC1 knockdown (KD) IPEC-J2 cells were infected with PEDV for 24 h. Total cell lysates were immunoprecipitated with the acetyl-lysine antibody, followed by immunoblotting with the STAT1, β-actin, and histone H3 antibodies; STAT1, HDAC1, and β-actin detection of total IPEC-J2 cell lysates were used as the input (left). (C) STAT1 acetylation as shown in panel B was analyzed and shown by the ac-STAT1/STAT1 ratio from 3 independent experiments.

We have recently reported the involvement of PEDV infection in the upregulation of protein acetylation due to reduced HDAC1 expression as a result of PEDV N protein binding and inactivation of the Sp1 transcriptional activity (25). Here, we further show that PEDV infection did enhance the acetylation of histone H3/H4 and a 100-kDa protein, similar to the effect of HDAC1 and -3 inhibitor MS-275 (Fig. 6A, left). Since STAT1 showed the same molecular weight (MW) at 100 kDa (Fig. 6A, right), we speculated whether STAT1 acetylation could be enhanced by PEDV and HDAC1 inhibition. Thus, IPEC-J2 cells with HDAC1-knockdown (IPEC-J2^HDAC1-KD^) and the wild-type cells were then infected with PEDV, followed by acetylated lysine immunoprecipitation. Figure 6B and C shows enhanced acetylation of STAT1 in PEDV-infected wild-type (WT) cells and HDAC1 knockdown cells. Because PEDV inhibited HDAC1 expression as we reported recently (25), we believe that PEDV possibly promotes STAT1 acetylation via inhibition of HDAC1.

### PEDV-enhanced acetylation blocks STAT1 from activation.

**FIG 7:**
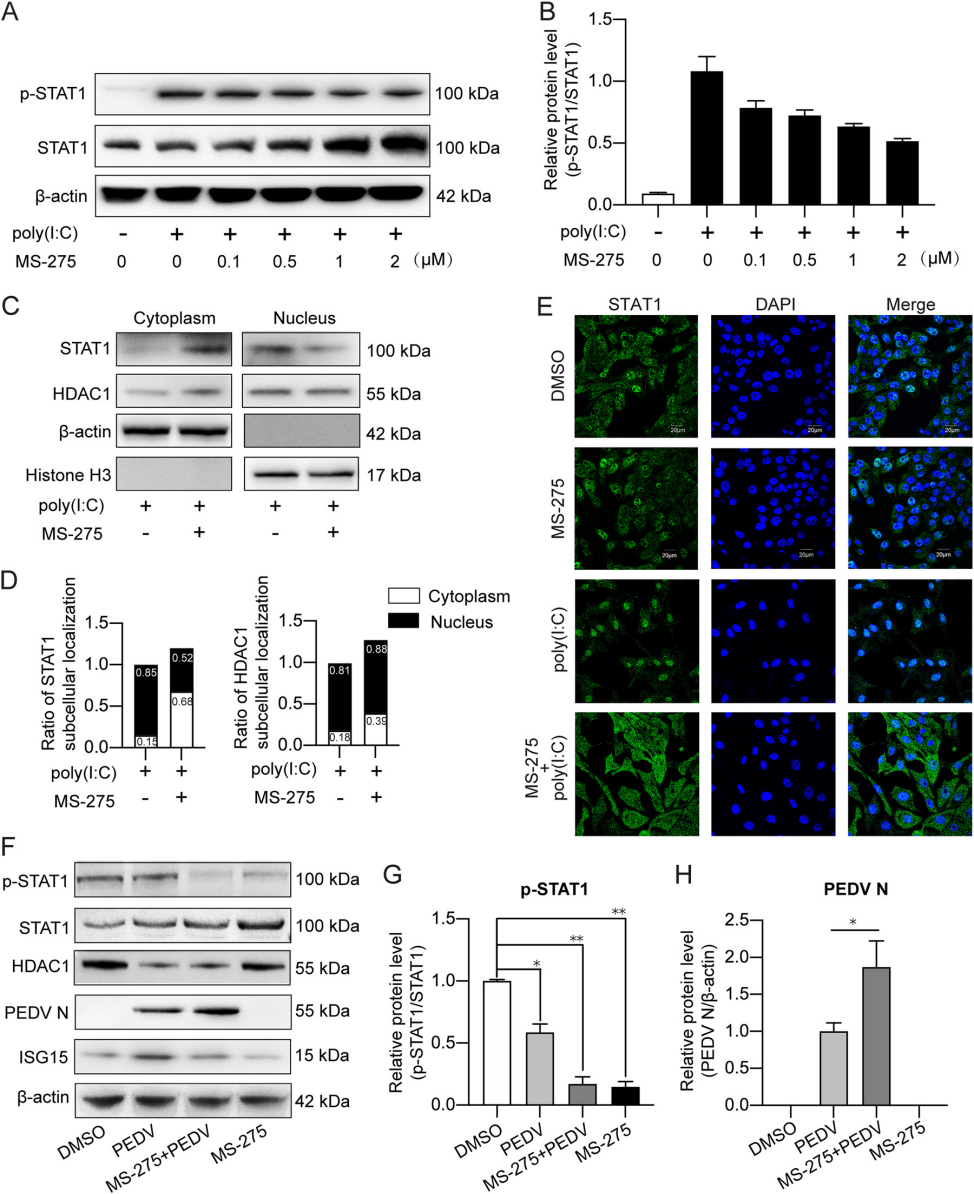
PEDV-enhanced STAT1 acetylation diminishes its phosphorylation. (A and B) STAT1 phosphorylation in IPEC-J2 cells affected by MS-275 treatment analyzed by Western blotting. The cells were pretreated with MS-275 at a concentration gradient, followed by poly(I·C) transfection for 24 h; MS-275 was maintained during the whole process. Total cell lysates were employed for SDS-PAGE followed by Western blotting using phosphorylated-STAT1 and total STAT1 antibodies; β-actin was used as the loading control. (C and D) STAT1 and HDAC1 subcellular localization measured and analyzed by cytoplasmic and nuclear fraction after poly(I·C) transfection and MS-275 treatment. The quality of cytoplasmic and nuclear fractionation was symbolized by β-actin and histone H3, respectively. (E) STAT1 cellular distribution was visualized by immunocytochemistry. The nuclei are shown by DAPI staining. (F) STAT1 phosphorylation in IPEC-J2 cells both treated with MS-275 and infected with PEDV. The cells were pretreated with 1 μM MS-275 for 1 h followed by PEDV infection. After 24 h postinfection, phosphorylated STAT1 together with total STAT1, PEDV N, and HDAC1 expression were measured by Western blotting. (G) The STAT1 phosphorylation and PEDV N expression were further obtained by densitometric analysis from three independent experiments. The results of panels G and H are shown as means ± SD. *, *P < *0.05; **, *P < *0.01.

Impaired phosphorylation and enhanced acetylation of STAT1 during PEDV infection prompted us to explore the relationship between these two posttranslational modifications. We transfected the IPEC-J2 cells with poly(I·C) together with MS-275 treatment at a concentration gradient from 0.1 to 2 μM. As shown in Fig. 7A and B, p-STAT1 was highly induced by poly(I·C). Nonetheless, MS-275 treatment decreased the ratio of p-STAT1 to total STAT1 protein in a concentration-dependent manner. We also analyzed STAT1 subcellular localization by nuclear and cytoplasmic extraction and immunofluorescence. Figure 7C to E corroborate that HDAC1 inhibition by MS-275 perturbed distribution of STAT1 in favor of the cytoplasmic compartment, similar to the findings in the PEDV-infected and poly(I·C)-stimulated cells (Fig. 5D to F), suggesting that enhanced acetylation of STAT1 affects its nuclear translocation.

To investigate if PEDV utilizes STAT1 acetylation to suppress its activation, IPEC-J2 cells were treated with 1 μM MS-275 followed by PEDV infection. PEDV infection, MS-275 treatment, and their combination downregulated the p-STAT1 proportion (Fig. 7F and G). MS-275 inhibition of HDAC1 led to increased PEDV N expression (Fig. 7F and H). It is evident from the above-described results that PEDV takes advantage of protein acetylation to block STAT1 activation in favor of its replication.

### PEDV inhibits STAT1 activation via its N protein.

**FIG 8:**
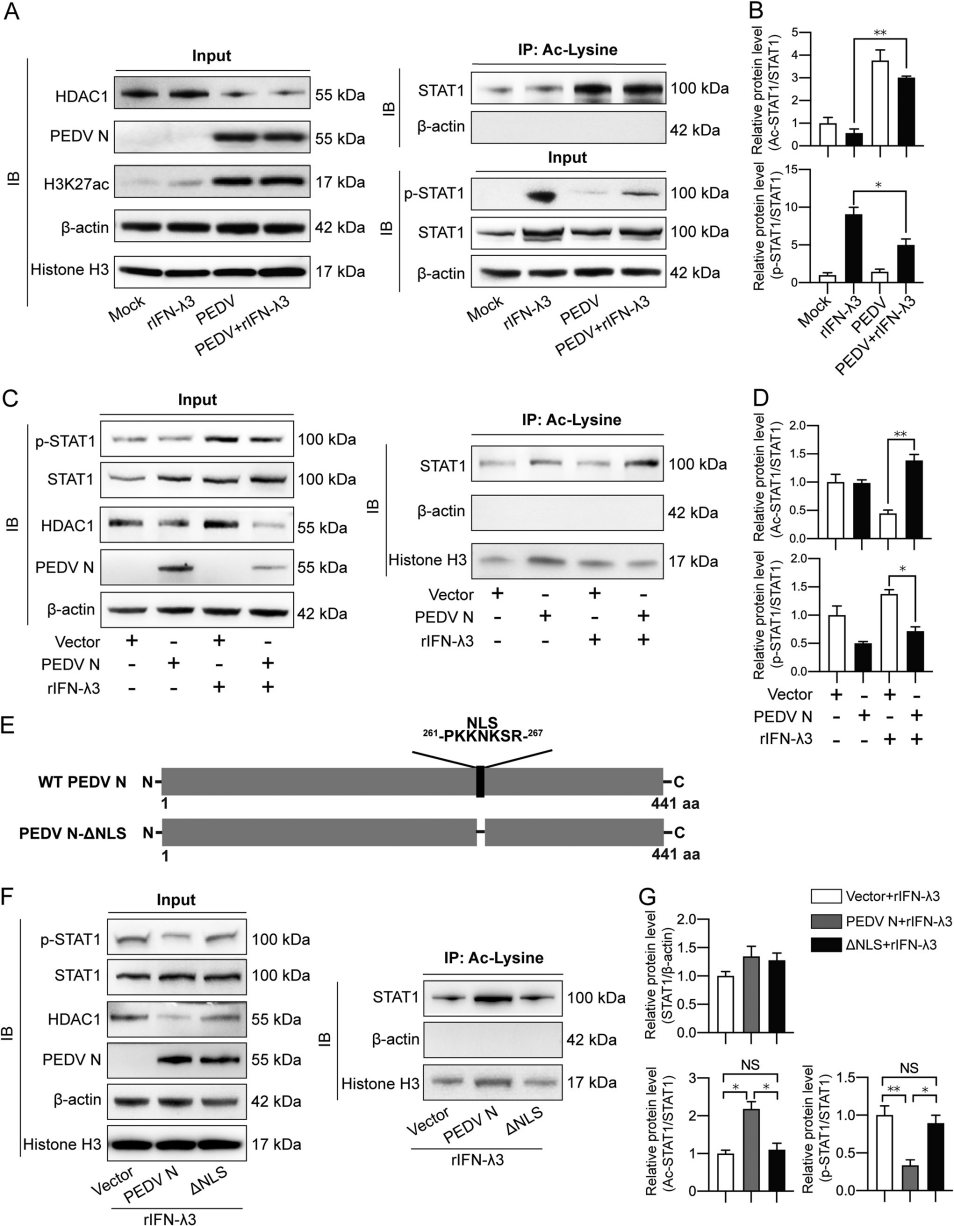
PEDV utilizes its N protein to induce STAT1 acetylation and suppress STAT1 phosphorylation. (A) STAT1 expression, acetylation, and phosphorylation were analyzed from the IPEC-J2 cells infected with PEDV followed by treatment of rIFN-λ3. The cells were first infected with PEDV at an MOI of 1; after 4 h absorption, the cells were treated with rIFN-λ3 to the concentration of 50 ng/mL for another 20 h, after which total protein was extracted. Acetylated STAT1 level was measured by acetylated lysine antibody enrichment followed by immunoblotting using total STAT1 antibody. STAT1 phosphorylation was detected using the phosphorylated STAT1 antibody at Y701. Expression of the other proteins was immunoblotted by specific antibodies mentioned in Materials and Methods. Histone H3 and β-actin were shown as loading controls. (B) STAT1 acetylation and phosphorylation as shown in panel A were analyzed and represented by the ac-STAT1/STAT1 ratio and p-STAT1/STAT1 ratio, separately. (C and D) STAT1 phosphorylation and acetylation were analyzed after PEDV N overexpression followed by rIFN-λ3 treatment, as the same procedures with panel A. (E) Schematic diagram showing NLS-deleted PEDV N mutant construction. (F and G) STAT1 expression, acetylation, and phosphorylation were analyzed from the IPEC-J2 cells exogenously expressed with WT and ΔNLS PEDV N followed by treatment of rIFN-λ3. The results of panels B, D, and G were obtained from 3 independent experiments of panels A, C, and F, respectively, and are shown as means ± SD. *, *P < *0.05; **, *P < *0.01.

To further illustrate that PEDV suppresses STAT1 phosphorylation by downregulating HDAC1 expression, we infected IPEC-J2 cells with PEDV 4 h before rIFN-λ3 treatment. As shown in Fig. 8A and B, rIFN-λ3 strongly induced STAT1 expression and phosphorylation, while PEDV severely blocked STAT1 phosphorylation induced by exogenous IFN-λ3. Moreover, downregulation of HDAC1 expression by PEDV upregulated STAT1 acetylation but suppressed STAT1 phosphorylation, implying that PEDV inhibits STAT1 activation by manipulating its acetylation over phosphorylation.

We have previously demonstrated that the PEDV N protein uses its nuclear localization sequence (NLS; ^261^-PKKNKSR-^267^) to enter into the nucleus to block HDAC1 transcription by binding to Sp1 (25). We wanted to know whether PEDV inhibits STAT1 activation by promoting STAT1 acetylation due to N protein-mediated suppression of HDAC1. PEDV N overexpression was performed in the IPEC-J2 cells followed by rIFN-λ3 treatment. PEDV N protein increased acetylation and impaired phosphorylation of STAT1 in response to IFN-λ3 activation (Fig. 8C and D), similar to PEDV infection (Fig. 8A). However, NLS deletion of PEDV N (ΔNLS) (Fig. 8E) reversed the effects on STAT1 by the wild-type N protein, i.e., reduced acetylation but increased phosphorylation (Fig. 8F and G). It is noteworthy that the PEDV N protein did not affect STAT1 acetylation without exogenous IFN-λ3 treatment (Fig. 8C and D), while PEDV infection did significantly (Fig. 8A and B). As PEDV itself was previously reported to induce IFN-λ expression in Vero E6 cells (32), it is possible that the N protein might play a role in regulating the STAT1 acetylation-phosphorylation balance at the whole-virus level, though further study is required.

### HDAC1 downregulation by PEDV infection impairs ISG expression.

**FIG 9:**
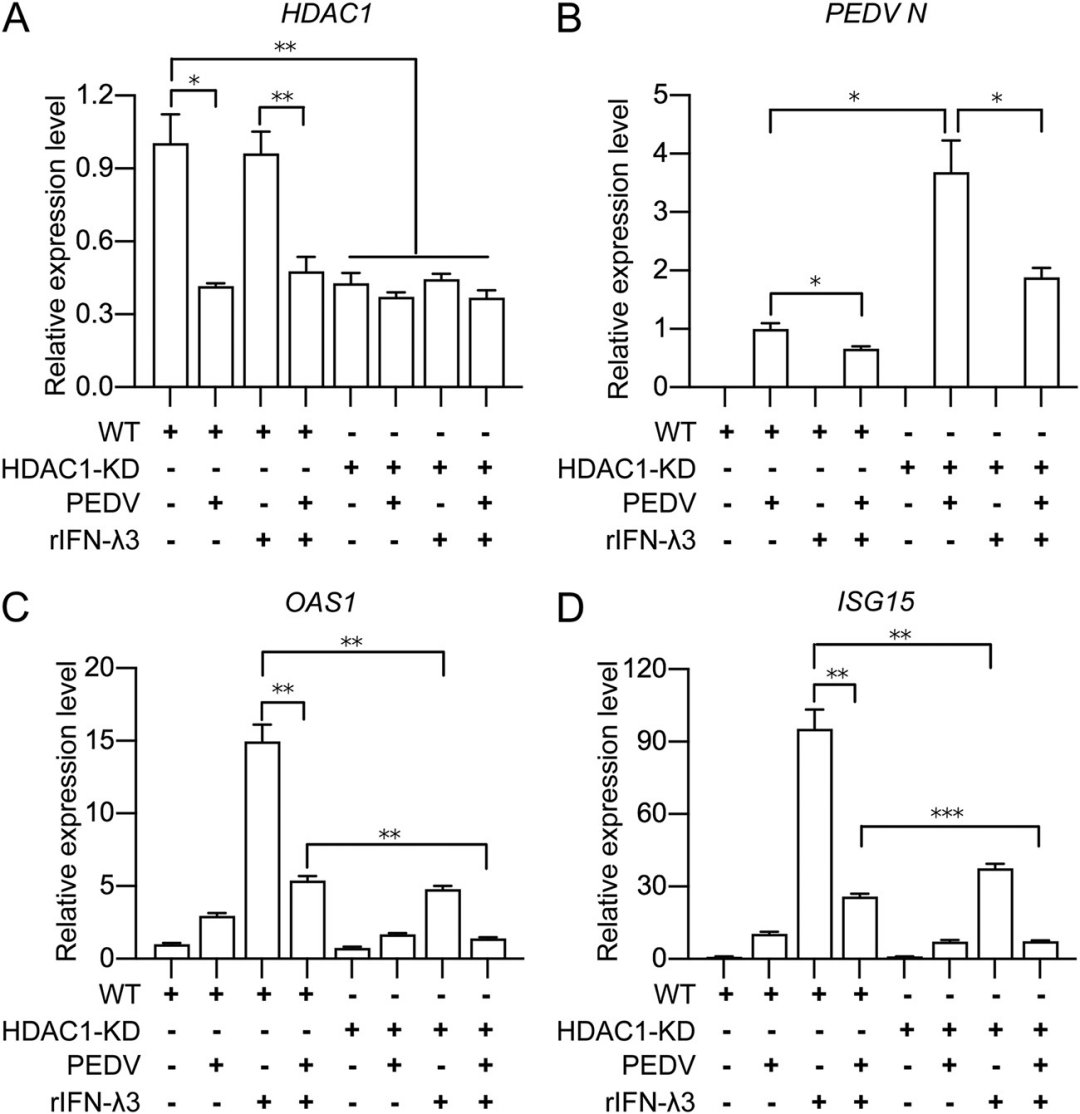
PEDV inhibits IFN-λ3-induced ISG expression in an HDAC1-dependent manner. (A to D) The wild-type IPEC-J2 cells and HDAC1 knockdown cells were infected with PEDV (MOI = 1) followed by rIFN-λ3 treatment (50 ng/mL) as shown in Fig. 8. The obtained total mRNA was used to detect transcription levels of *HDAC1* (A), PEDV N (B), *OAS1* (C), and *ISG15* (D) by qRT-PCR; these genes were normalized to *GAPDH*. Data of panels A to D were shown as means ± SD from 3 independent experiments with the error bars representing SD. *, *P < *0.05; **, *P < *0.01; ***, *P < *0.001.

Because the above-described results have confirmed that PEDV inhibits STAT1 activation in an HDAC1-dependent manner, we wondered whether HDAC1 is involved in IFN-λ-induced antiviral ISG expression and if PEDV evasion of the host antiviral immunity is HDAC1 dependent. In our previous study, we found that PEDV inhibits *OAS1* and *ISG15* expression via blocking Sp1-mediated transcriptional activation of *HDAC1* (25). Thus, we examined the effects of HDAC1 knockdown on the transcriptional changes of ISGs and PEDV N in the cells with or without IFN-λ3 activation. Figure 9A shows that PEDV infection inhibited *HDAC1* expression to a degree similar to HDAC1 knockdown (columns 2 and 5). HDAC1 knockdown led to increased transcription of PEDV N, while IFN-λ3 treatment reduced its transcription either in HDAC1 knockdown cells or the wild-type cells (Fig. 9B), suggesting that HDAC1 acts as an anti-PEDV factor similar to IFN-λ3. HDAC1 knockdown or PEDV infection alone significantly suppressed IFN-λ3-induced *OAS1* and *ISG15* expression, while the combination of HDAC1 knockdown and PEDV infection showed more suppressive effects (Fig. 9C and D). Therefore, we propose that PEDV evasion of the host cell antiviral response is HDAC1 dependent because PEDV mediated inhibition of both *HDAC1* (Fig. 9A) and *OAS1* and *ISG15* (Fig. 9C and D) expression in response to IFN-λ3 treatment, and there was enhanced suppression of *OAS1* and *ISG15* transcription in PEDV-infected and HDAC1 knockdown cells (Fig. 9C and D).

## DISCUSSION

**FIG 10:**
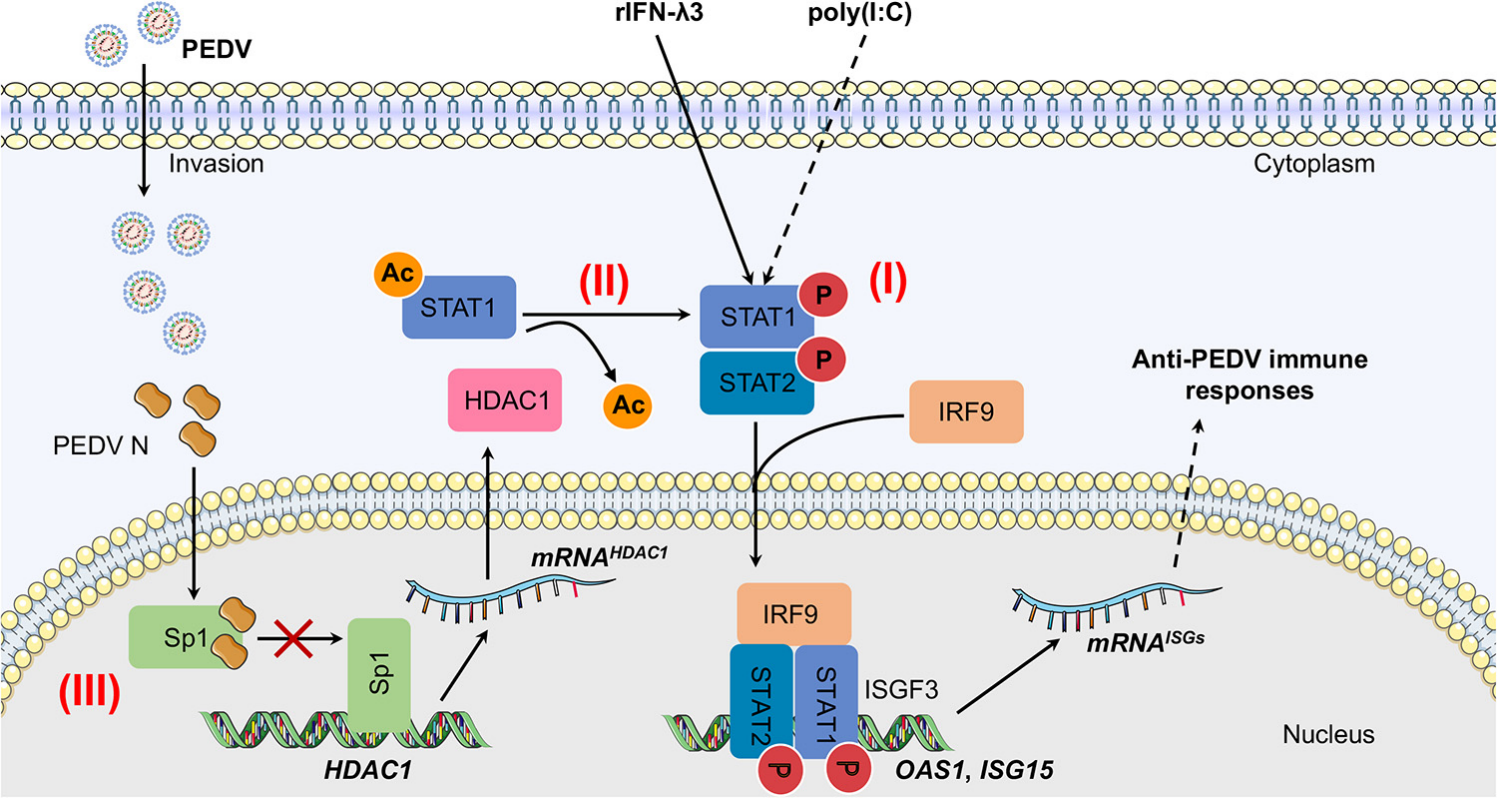
PEDV antagonizes the expression of antiviral ISG15 and OAS1 by promoting STAT1 acetylation with subsequent reduction of its phosphorylation and nuclear translocation. (I) JAK-STAT1 signaling is directly activated by type III IFN stimulation (solid line), while it is indirectly activated by poly(I·C) (broken line). The phosphorylated STAT1 forms a heterodimer with activated STAT2, which further associates with IRF9 to form the ISGF3 complex. The complex is then translocated into the nucleus, where it binds to the target promoter and activates transcription of *OAS1* and *ISG15*. (II) HDAC1 utilizes its deacetylase activity to facilitate STAT1 phosphorylation. (III) PEDV infection inhibits HDAC1 expression through the interaction of its N protein with Sp1, resulting in enhanced STAT1 acetylation. The acetylated STAT1 fails to be phosphorylated and is prevented from entering the nucleus. Consequently, *ISG15* and *OAS1* transcription is impaired. Therefore, PEDV evades the antiviral effect of ISGs by preventing STAT1 activation as a result of viral N protein-mediated suppression of the Sp1 transcriptional activity and decreased expression of HDAC1.

The expression of type I and III IFN genes is distinctly regulated following pathogen-associated molecular pattern recognition by unique pattern recognition receptors (PRRs), while IFN-mediated signaling cascades are shaped by the strength of the ligand-receptor interactions, abundance of cell surface receptors, and availability of transcriptional regulators (33). Our initial experiment indicated that PEDV infection suppressed expression of *OAS1* and *ISG15* induced by both poly(I·C) and IFN-λ3 (Fig. 1), suggesting that the virus might inhibit the molecules downstream of the IFN receptors, such as the JAK-STAT pathway. In our recent publication, PEDV was found to utilize its N protein to bind and suppress the transcription factor Sp1, leading to reduced HDAC1 expression (25). Although STAT signaling is known to be regulated by histone acetyltransferase (HAT)-HDAC interplay (34), it remains unknown if STAT1 activation is regulated by HDAC1. By inhibition of HDAC1 via gene knockdown or chemical inhibition or activation of STAT1 with poly(I·C) or IFN-λ3 in PEDV-infected IPEC-J2 cells or cells expressing the N protein, we clearly demonstrate that PEDV deploys its N protein to suppress STAT1 activation and expression of *OAS1* and *ISG15* by induction of STAT1 acetylation over phosphorylation as a result of downregulation of HDAC1 by the PEDV N protein, as we have reported elsewhere (25) (Fig. 10).

Acetylation-mediated inhibition of phosphorylation was previously reported in IFN-α treated 293T cells: STAT1 activation is strikingly inhibited by CBP-dependent acetylation and also influenced by HDAC3 and T-cell protein tyrosine phosphatase 45 (TCP45) (35). HDAC inhibitors also downregulate IFN-γ-induced STAT1 phosphorylation in both primary and transformed cells, and the acetylated STAT1 binds to TCP45, which, in turn, inhibits STAT1 activation via dephosphorylation (36). The authors proposed a phosphorylation-acetylation switch in posttranscriptional regulation of STAT1 in affecting IFN signaling (35, 37). However, this was challenged by a following report showing that HDAC inhibition had no influence on STAT1 acetylation and subsequent phosphorylation (38). This controversy might be attributed to the antibodies used, e.g., pan-acetylated lysine antibodies versus site-specific antibodies, as well as the localization of key acetylation sites (Lys 410 and Lys 413) of STAT1 (39). Here, we show that PEDV infection or its N protein expression tilted STAT1 toward acetylation over phosphorylation, thus blocking STAT1 activation and inhibiting its downstream ISG expression. Further study is warranted to investigate which lysine residue(s) are targeted by HDAC1 in PEDV-infected cells and how STAT1 acetylation in this particular residue affects its phosphorylation or its heterodimer formation with STAT2 and IRF9.

The type III IFN system dominates the antiviral innate immunity of intestinal epithelial cells, and JAK-STAT signaling is an evolutionary conserved antiviral pathway in regulating type III IFN responses (16, 40). Of the 7 STAT members (STAT1, -2, -3, -4, -5a, -5b, and -6), STAT1 is one of the most widely reported proteins in antiviral immunity due to its conservation and a large number of its regulated genes (41). By forming a homodimer or heterodimer with STAT2 induced by IFNs, STAT1 could mount antiviral innate immune responses. For instance, STAT1 is necessary for IFN-λ-induced ISGs expression and inhibition of hepatitis C virus (HCV) replication (42). With SARS-CoV infection, STAT1 plays an important role in protecting the host from severe sickness independent of IFN receptor-mediated signaling (43). Here, we show that STAT1-mediated antiviral signaling is functional against PEDV. Of the antiviral ISGs examined, PEDV seemed to inhibit IFN-λ3-induced expression of *OAS1* and *ISG15*, but not *IFITM1* and *MX1* in the IPEC-J2 cells. It is probably because these genes are differentially regulated, *IFITM1* by IFN-γ (44) and *MX1* by IFN-τ (type I IFN) (45). In our recent study, HDAC1 inhibition by MS-275 treatment was found to repress transcription of *ISG15* and *OAS1* (25). These findings indicate that PEDV infection inhibits IFN-λ-induced ISG expression through the HDAC1-STAT1 signaling. Because IFN-λ treatment or PEDV infection could upregulate STAT1 expression in IPEC-J2 cells (Fig. 8A), STAT1 itself acts as an ISG in the PEDV-IFN signaling interplay, affecting a series of downstream molecular events (46).

PEDV has developed a number of tactics to evade host innate immune responses, and a number of PEDV proteins were reported to suppress IFN responses (47, 48). For instance, PEDV nonstructural protein 1 (nsp1) interacts with type III IFN signaling activation by reducing the level of peroxisomes in IPEC-DQ cells (16). PEDV nsp2 targets the F-box and WD repeat domain-containing 7 protein for degradation via the ubiquitin-proteasome system, finally hampering the expression of ISGs (49). To antagonize the host IFN response, PEDV also utilizes its nsp15 to downregulate transcription of *TBK1* and *IRF3* (50). The N protein targets the host TBK1 by direct interaction, leading to inhibition of IRF3 activation and IFN-β production (9). We recently found that PEDV significantly downregulates the transcription level of several HDAC members in IPEC-J2 cells, especially HDAC1 (25). In this study, we demonstrated a novel immune evasion strategy that PEDV employs its N protein to downregulate HDAC1, leading to inhibition of STAT1 activation by tilting acetylation over phosphorylation to evade antiviral IFN immune responses to benefit its replication. In hepatitis C virus infection, the viral core protein increased STAT1 acetylation and blocked its phosphorylation by decreasing the transcription level of HDAC4, resulting in reduced host immune responses to IFN-α stimulation (51). From the perspective of PEDV, since the nuclear localization of the PEDV N protein is crucial for the interaction with Sp1 and the downstream HDAC1-STAT1 signaling, it is also valuable to employ the recombinant PEDV-mutating NLS for a better understanding of PEDV immune evasion in the future.

Although we confirmed that PEDV infection suppresses STAT1 phosphorylation and its translocation into the nuclei by nuclear-cytoplasmic separation and immunofluorescence assay (Fig. 5), it is worth mentioning that STAT1 was also blocked in the cytoplasm of the uninfected cells (Fig. 5F). Virus infection is known to change the biological processes of both infected and the surrounding cells. Libraty et al. showed that both the infected and uninfected surrounding dendritic cells could be activated by the live dengue virus to produce TNF-α and IFN-α (52). Also, Schmidt et al. observed significant cellular DNA synthesis of hundreds of uninfected cells during herpes simplex virus infection, indicating a paracrine effect (53). Taken together, the uninfected surrounding cells could also be activated during virus infection.

In conclusion, we have demonstrated a novel immune evasion mechanism that PEDV makes use of its N protein to manipulate the reciprocal relationship of STAT1 acetylation and phosphorylation, which is to enhance STAT1 acetylation due to downregulation of HDAC1 with subsequent blockage of its phosphorylation and nuclear translocation to dampen the antiviral IFN signaling in favor of viral replication. Our findings contribute to a better understanding of the PEDV-host interaction as part of pathogenetic mechanisms of coronaviruses.

## MATERIALS AND METHODS

### Cell lines and virus preparation.

The porcine intestinal epithelial cell line (IPEC-J2), IPEC-J2 cells with stable HDAC1 knockdown (IPEC-J2^HDAC1-KD^), and Vero E6 cell line were stocked in our laboratory. The IPEC-J2 cells were maintained in Dulbecco’s modified Eagle’s medium-F12 (DMEM-F12; Gibco) with 10% fetal bovine serum (FBS; Gibco), 100 U/mL penicillin, 0.1 mg/mL streptomycin, and 0.25 mg/mL amphotericin B (Gibco) at 37°C with 5% CO_2_. The IPEC-J2^HDAC1-KD^ cells were previously produced in our laboratory by using the CRISPR/Cas9 system (25) and selection with 10 μg/mL of puromycin and stored in liquid nitrogen for further use. The Vero E6 cells were cultured in DMEM supplemented with 10% FBS under the same conditions. The PEDV strain ZJ15XS0101 maintained in our laboratory was isolated from the porcine intestines of a diseased pig and was propagated and titrated in Vero E6 cells (54).

### PEDV infection and treatments with recombinant IFNs or chemical inhibitors.

To perform the experimental infection, IPEC-J2 cells were first seeded into wells of a 6-well plate and grown to 60% confluence, after which the cells were infected with PEDV at a multiplicity of infection (MOI) of 1. After adsorption for 4 h at 37°C and 5% CO_2_, the inoculum was removed, washed with sterile phosphate-buffered saline (PBS), and replaced with PEDV maintenance medium (DMEM with 4 μg/mL of trypsin). Total RNA and proteins were extracted at proper time points with the method described below.

Commercial recombinant IFN proteins (rIFNs) used for STAT1 activation include recombinant human IFN-alpha 1 protein (rIFN-α1; RP00011; ABclonal, China), active recombinant human IFN-gamma protein (rIFN-γ; RP01038; ABclonal), and active recombinant human IFN-lambda 3/IL-28B protein (rIFN-λ3; RP00219; ABclonal) were tested as endotoxin free and of high protein purity. According to different study purposes, the IPEC-J2 cells were treated with rIFNs in two ways, (i) to detect IFN signaling/STAT1 activation in anti-PEDV immune responses, the IPEC-J2 cells were pretreated with 50 ng/mL of rIFNs (rIFN-α1, rIFN-γ, rIFN-λ3) for 30 min, followed by PEDV infection (MOI = 1); and (ii) to investigate PEDV functions in regulating STAT1 modification and ISG expression, the IPEC-J2 cells were inoculated with PEDV at the MOI of 1 and incubated for 4 h to allow virus adsorption, and the cells were then treated with 50 ng/mL of rIFN-λ3 for next 20 h. Afterward, the cells were collected for RNA extraction and cell lysis.

An HDAC1- and HDAC3-specific inhibitor, Entinostat (also named MS-275; Selleck), was used to suppress HDAC1 activity and enhance protein acetylation level. The IPEC-J2 cells were pretreated with 1 μM MS-275 for 1 h before PEDV infection as described above or poly(I·C) transfection as described below. Fludarabine (also termed FaraA; catalog no. NSC118218; Selleck) was used as an inhibitor of STAT1 expression in IPEC-J2 cells. Briefly, the IPEC-J2 cells grown to 60% confluence were treated with 1 μM FaraA for 1 h, followed by PEDV infection at a multiplicity of infection (MOI) of 1 for 24 h. The PEDV-infected or poly(I·C)-stimulated IPEC-J2 cells were collected for RNA extraction and cell lysis, as mentioned below.

### Construction of recombinant plasmids and transfection.

The recombinant pCMV-based plasmid with an HA tag for porcine STAT1 overexpression was constructed by inserting the full-length *STAT1* sequence (GenBank accession no. NM_213769) cloned from the IPEC-J2 total RNA using a reverse transcription-PCR (RT-PCR) method and the ClonExpress II one-step cloning kit (Vazyme, Nanjing, China) with a pair of specific primers (*pCMV-STAT1F* and *pCMV-STAT1R*) (Table 1). The recombinant plasmid was confirmed by sequencing, qRT-PCR, and Western blotting.

**TABLE 1:** Primers used in this study

Primer	Direction^*a*^	Sequence (5′–3′)
Real-time PCR		
STAT1	F	GGGTGTATTGTGGGCTTTA
	R	AGGTTCGCCTCCGTTCT
STAT2	F	CCCCATCGGACCAGACA
	R	TGCCGCAGGTGAAACAA
STAT3	F	GATGTTCGCAAGCGTGTC
	R	GGCAGATGTTGGGAGGG
STAT4	F	TTGGATTGATGGGTATG
	R	GTCAGCGAATGGTAGAG
STAT5a	F	GCGGTGCCTGACAAAGT
	R	CAGCCTGGTAAGTTCTCCCT
STAT5b	F	GACCGTCGTGGAGCAGA
	R	AAAGCATTGTCCCAGAGG
STAT6	F	GACGCAGACCAAGTTTCAG
	R	GGCAGAGCAGCAGTTCC
IRF9	F	CAGCAACAGCCCTGAGTC
	R	GTGCCCGTCGTAGATGAA
HDAC1	F	ATGAGGAGGGAGAAGGTG
	R	GGTTGTGGGATAAAGACG
PEDV N	F	CGGAACAGGACCTCACGCC
	R	ACAATCTCAACTACGCTGGGAAG
IFITM1	F	TGCCTCCACCGCCAAGT
	R	GTGGCTCCGATGGTCAGAAT
MX1	F	AATGAGAGAGGAGATGTGTG
	R	TGAACCCAGATTGGAAGC
OAS1	F	GCCTGTGATTCTGGACCCGGCTGA
	R	CGACACCTTCCAGGATCCCACCG
ISG15	F	ATGGGTAGGGAACTGAAGGT
	R	CAGACGCTGCTGGAAGG
GAPDH	F	CACTGAGGACCAGGTTGTGTCCTGTGAC
	R	TCCACCACCCTGTTGCTGTAGCCAAATTC
Vector construction		
pCMV-STAT1	F	GAGATCTGCCTCGAGATGTCCCAGTGGTATGAGCTTCAG
	R	AACATCGTATGGGTAGTCAAGGTTCATAGTTCCAGAGAACTTGTTC
siSTAT1	F	GCGUAACCUUCAGGAUAAUTT
	R	AUUAUCCUGAAGGUUACGCTT
pCMV-N	F	GACGATGACAAGCTTGCGGCCGCTATGGCTTCTGTCAGTTTTCAGGA
	R	CAGGGATGCCACCCGGGATCCTTAATTTCCTGTATCGAAGATCTCGT
pCMV-N ΔNLS	F	GGCAAAAATACAGCCACTTCGAAAGAACGTGACC
	R	TTCGAAGTGGCTGTATTTTTGCCGCTGCTGTCAG

a F, forward, R, reverse.

The recombinant plasmid for STAT1 overexpression, predesigned small interfering RNA (*siSTAT1F* and *siSTAT1R*) for STAT1 knockdown, and poly(I·C) were transfected, where appropriate, into IPEC-J2 cells using Lipofectamine 2000 (Invitrogen) according to the manufacturer’s instructions.

### RNA extraction and quantitative RT-PCR.

After PEDV infection, chemical inhibition, small interfering RNA (siRNA), or eukaryotic expression vector transfection, the IPEC-J2 cells were lysed with TRIzol RNA isolation reagent (Thermo). Total RNA was then extracted with an RNA extraction kit (Bioteke, Beijing, China) following the manufacturer’s instructions. cDNA for expression analysis of target genes was synthesized using the HiScript II Q RT supermix for qPCR (+DNA wiper) (Vazyme) with the extracted total RNA as the template. The transcription levels of the PEDV N gene, ISGs, and different STAT genes were then analyzed by qRT-PCR with AceQ universal SYBR qPCR master mix (Vazyme) and specific RT primers (Table 1). The qRT-PCR profile was as 95°C for 10 min, 40 cycles of 95°C for 15 s, 58°C for 50 s, and 72°C for 2 s, and the melting curve obtained from 65°C to 95°C. Relative quantification of target genes was normalized to the reference gene, *GAPDH* (glyceraldehyde-3-phosphate dehydrogenase), and analyzed by the threshold cycle (2^−ΔΔ^*^CT^*) method as reported previously (55). The data were presented as means plus standard deviations (SDs). Significant differences were determined by Student’s *t* test, with a *P* value of <0.05 considered statistically significant and a *P* value of <0.01 of marked statistical significance.

### Cell lysis and Western blotting.

The IPEC-J2 cells infected with PEDV at appropriate MOIs or transfected with recombinant expression plasmids or siRNA were used for total protein extraction using the cell lysis buffer (Beyotime, China). The protein concentration of the whole-cell lysates in different treatments was measured by a bicinchoninic acid (BCA) protein assay kit (Thermo). Afterward, the heat-denatured protein samples were loaded onto 12% SDS-PAGE gels for protein fractionation followed by blotting onto a 0.22-μm polyvinylidene difluoride membrane (PVDF; Millipore). The proteins on the blots were then probed by specific primary and secondary antibodies, including rabbit anti-STAT1 (CST), rabbit anti-phospho-STAT1 at Y701 (ABclonal), rabbit anti-acetyl-lysine (Abcam), rabbit anti-acetyl-H3-K27 (ABclonal), rabbit anti-HDAC1 (ABclonal), rabbit anti-ISG15 (Beyotime), mouse anti-β-actin (Thermo), mouse anti-histone H3 (Beyotime), rabbit anti-HA tag (CST), mouse anti-PEDV N (monoclonal antibody reserved in our laboratory), goat anti-mouse IgG (Invitrogen) and goat anti-rabbit IgG (Invitrogen). The target protein bands on the membrane were developed using an ECL kit (Cyanagen, Italy) and were visualized by an imaging system (SageCreation, Beijing, China) for densitometric analysis of their relative levels of expression.

### Co-IP assay.

Since the antibody recognizing acetylated-STAT1 was not available, immunoprecipitation (IP) using the acetyl-lysine antibody was performed to detect STAT1 acetylation in the PEDV-infected cells. Briefly, after PEDV infection, poly(I·C) or rIFN-λ3 treatment, or chemical inhibition, the total protein was extracted using the same method as described above, after which the cell lysates were incubated overnight with 2 μg of acetyl-lysine antibody at 4°C. Subsequently, the acetyl-lysine antibody binding to the target proteins was captured by the protein A+G agarose (CST). All procedures were strictly operated at low temperatures (0 to 4°C) to avoid loss of the protein modification signal. Acetylted-STAT1 was detected by Western blotting using specific total STAT1 antibody as shown above.

### Immunofluorescence assay.

To visualize STAT1 subcellular distribution and activation, immunocytochemistry was performed. The IPEC-J2 cells were fixed by 4% paraformaldehyde and probed by rabbit STAT1 antibody after PEDV infection or poly(I·C) treatment for 24 h. The cells were labeled with Alexa Fluor 488 goat anti-rabbit IgG (Invitrogen) to recognize cytoplasmic and nuclear STAT1. To detect PEDV N protein distribution, the cells were probed by PEDV N mouse monoclonal antibody followed by labeling with Alexa Fluor 555 donkey anti-mouse IgG (Invitrogen). The nuclei were stained by 4′,6-diamidino-2-phenylindole (DAPI; Thermo). The subcellular location of STAT1 and PEDV N was analyzed by a confocal laser scanning microscope (IX81-FV1000; Olympus).
